# Ten Years of High and Intensive Care Within Psychiatry: Professionals' Reflections Upon Developments and Future Implications

**DOI:** 10.1111/inm.70120

**Published:** 2025-08-22

**Authors:** I. C. de Jong Isa, A. L. van Melle Laura, S. Gerritsen Sylvia, C. L. Mulder Cornelis, M. A. van den Hoven Mariëtte, Y. Voskes Yolande

**Affiliations:** ^1^ Department of Ethics, Law and Humanities Amsterdam University Medical Center Amsterdam the Netherlands; ^2^ GGZ Ingeest Amsterdam the Netherlands; ^3^ Department of Psychiatry, Epidemiological and Social Psychiatric Research Institute Erasmus University Medical Center Rotterdam the Netherlands; ^4^ Parnassia Psychiatric Institute Rotterdam the Netherlands; ^5^ Mental Healthcare Centre GGZ Eindhoven Eindhoven the Netherlands; ^6^ Tilburg School of Social and Behavioral Sciences, Tranzo Scientific Center for Care and Welfare Tilburg University Tilburg the Netherlands

**Keywords:** coercion, critical care, mental health, psychiatric department, hospital, quality of health care

## Abstract

Over the past decade, psychiatric wards across the Netherlands have worked in accordance with the High and Intensive Care model to reduce coercion and improve the quality of care. Securing implementation of the model within a challenging mental health care context has proven to be effective but complex in practice. Consequently, this study aimed to both gain insight into the process of implementing the High and Intensive Care model by drawing upon professionals' own reflections and provide recommendations for improving practice and the model. This is a national multicentre study that utilised qualitative methods. Data was collected by a total of 26 institutional and national group interviews on which thematic analysis was conducted. Staff turnover, coercion, collaboration with outpatient care, working methodically and the move from control to contact were found to be the key developments since the model's implementation. Future efforts should focus upon staff retention and acquisition to ensure continuity of care and safety. Ongoing evaluation of coercion is essential to further reduce coercion. Collaborations with outpatient care and other stakeholders should be intensified to promote effective care. Management support, reflexivity and a clear vision are required to strengthen methodical working and collaboration between wards to create uniformity of practice. Working in contact with patients ensures safety but requires time and behavioural change. Applying implementation science can support improvements in acute mental health care by systematically addressing barriers to change, promoting sustainable evidence‐based practices, and guiding the reduction of coercion. Further research into these barriers, including the exploration of non‐coercive strategies and stakeholder involvement, is needed to enhance High and Intensive Care implementation and similar practices.

## 1 Introduction

Over the last three decades, there have been numerous changes in mental health care within the Netherlands that have both improved and challenged mental health care organisations. First, increased emphasis upon reducing coercion within psychiatric care resulted in several initiatives designed to achieve precisely this goal; ultimately, these initiatives did not result in the establishment of a national policy (Voskes et al. 2011). Second, the lobbying for the reduction of psychiatric beds that had begun in the 1980s continued over the course of the last three decades, resulting in more patients being moved to outpatient care, either at home or in sheltered housing facilities (Ravelli 2006; van Veldhuizen 2007). Third, the primary focus of care shifted from a biomedical model to a recovery‐oriented approach to improve the quality of mental health care (Anthony 1993). Finally, in 2013, these developments culminated in a new nationwide approach to inpatient acute psychiatry: the high and intensive care (HIC) model (Voskes et al. 2021a).

## 2 Background

The HIC model is a contact‐based stepped‐care model that aims to stabilise people going through a psychiatric crisis in a brief amount of time, combining both a medical approach and a recovery approach (Anthony 1993). HIC care, rooted in care ethics (Voskes et al. 2021b), minimises coercion through contact‐based care, which strengthens provider‐patient relationships, effectively reduces coercive measures (Voskes et al. 2014) and increases patient satisfaction and medication adherence (Ruud and Friis 2022). The HIC model is grounded in the idea of fostering a healing environment for patients that takes into consideration the attitude of staff, hospitality, architecture and daytime activities. Stepped‐care models provide strategies for intensifying or ‘stepping up’ care: from a group‐based high care unit (HC), consisting of single‐patient rooms, shared living areas and a comfort room, to an intensive care unit (ICU), consisting of large single‐patient rooms where patients receive one‐on‐one care (Voskeset al. 2021a). When such units are insufficient and cannot ensure patients' safety, a high‐security room (HSR) is used (Voskes et al. 2021a). The HSR is a confined area designated exclusively for emergency situations, equipped with private sanitation and smart home technology to maximize patient autonomy. Its use is considered a coercive intervention of last resort, requiring constant supervision and intended to be applied for the shortest possible duration. In 2013, the HIC model was published in the HIC workbook (van Mierlo et al. 2013), including the HIC monitor, a scoring instrument used to test HIC model fidelity (van Melle et al. 2019).

In 2013, all acute admission units in the Netherlands were rebranded as HIC and began implementing the model independently, utilising their own funding. According to implementation science, the uptake of evidence‐based practices in health care requires research to identify implementation barriers and enablers, behavioural theories and frameworks to close the evidence‐practice gap (Handley et al. 2016). Previous research on what motivated professionals to implement HIC indicated these facilitators and barriers (van Melle et al. 2021a). Facilitators that were identified included clear leadership, involving staff, phased implementation, setting achievable goals, positive feedback, celebrating successes, training and reflection. On the other hand, barriers encompassed a lack of knowledge, lack of formal organisational support, facilities (e.g., not having an ICU), budget and time, resistance to change, shortage of staff, flex workers and perceived shortcomings of the HIC standards. The same researchers found that whilst higher HIC model fidelity was associated with less coercion, the model fidelity amongst HIC wards differed greatly (van Melle et al. 2021b). Parallel to the implementation of HIC, the context of mental health care within the Netherlands changed. In 2020, the Dutch Compulsory Care Act came into operation (Ministerie van Volksgezondheid, Welzijn en Sport 2020) stating that coercion can only be used as a last resort, in the least invasive form, for the shortest amount of time. Alongside this, the ongoing reduction in bed capacity inevitably increased pressure on the remaining (acute) inpatient clinics (Segeren et al. 2022), further exacerbated by the national shortage of mental health professionals (Boumans et al. 2023).

It is widely acknowledged that care for individuals with severe mental illness, who experience a crisis situation, sometimes needs to be intensified. In the Netherlands, care has taken shape through the HIC model. At the same time, international initiatives, particularly in Europe, seek to provide high‐quality inpatient care while reducing coercion, reflecting a global push for humane, effective approaches despite resource challenges. Considering the challenging context, it is legitimate to ask how professionals have managed to implement an intensive care model. Enhancing our understanding of this can help in terms of improving HIC practice but also assist others in the implementation of their caring models within psychiatry. Therefore, this study set out to address the following research question: how do professionals themselves reflect upon the developments that have taken place over the past 10 years with respect to working with the HIC model, and what are the implications of these professionals' reflections for improving both the HIC model and current practice?

## 3 Methods

### 3.1 Design

For this multicentre study, qualitative research was conducted, specifically semi‐structured group interviews at the institutional level and focus groups at the national level with mental health professionals. This two‐stage method was chosen to explore and compare developments across the different HICs in order to address the first part of the research question; and to interpret key findings from a collective perspective to address the second part.

### 3.2 Wards and Participants

All of the mental health institutions across the Netherlands were approached to take part in the study, with 24 ultimately choosing to do so. Each institution selected one HIC ward to participate. Selection criteria were treating adult psychiatric patients (18+), and being equipped with an HSR. For the institutional group interviews, contact persons were asked to each recruit 6–7 healthcare professionals from their HIC, favourably all from different disciplines, to ensure diversity (Gray 2018). No minimum level of HIC experience was required, for new perceptions were considered valuable. The first author (IdJ) and the participating HICs were not yet acquainted with each other; four of the other authors were familiar with some of the participants through previous research. In total, 23 institutional group interviews were conducted, as one ward withdrew before participation due to organisational issues. The interviews consisted of 3–12 participants (average of six) each (Table 1). For the national focus groups, contact persons of the 24 institutions were asked to each recruit 2 to 3 professionals per ward, following the same selection criteria as for the institutional group interviews. Finally, 3 national focus groups were held, comprising 10–14 participants each, from 21 wards (Table 1).

**TABLE 1:** Professions of participants.

Profession	Institutional group interviews (*N* = 138)	National focus groups (*N* = 33)
(Coordinating) Nurse	67	22
Social worker	19	2
Psychiatrist	13	2
Activity counsellor	10	1
Team leader	9	2
Doctor (not) in training	6	—
Psychologist	5	—
Nurse practitioner	5	—
Peer counsellor	4	4

### 3.3 Data Collection

Data collection took place in the Netherlands between November 2022 and June 2023. During initial meetings with the ward contact persons, IdJ inductively gathered themes—without their explicit awareness—which informed the topic list for the subsequent group interviews (Table 2). Subsequently, 23 semi‐structured group interviews were conducted in the respective wards, providing a familiar setting for multidisciplinary teams to discuss developments within their ward. These interviews lasted 90 min each and were led by IdJ; three alongside fellow researchers. During the interviews, each participant received a set of 20 theme cards, ranked the top five most influential developments in their HIC work over the previous decade, and selected one least influential theme. Participants explained and compared their choices. The ranking process was used to help participants organise their thoughts and prioritise what they truly considered important. Afterwards, we carried out a preliminary analysis of the group interviews to identify the most prominent or differing themes. These themes served as a topic guide for the subsequent national focus groups (Table 3), which were designed to facilitate dialogue between HICs and to contextualise and deepen our understanding of the previous findings. Given the relatively large number of themes and our intention to explore them in depth, we divided the themes across the focus groups. There were three national focus groups held at the Amsterdam University Medical Center, led by LvM (second author), SG (third author) and YV (sixth author), and lasted 90 minutes. All group interviews and focus groups were audio recorded and transcribed verbatim.

**TABLE 2:** Topic guide institutional group interviews.

Instruction and themes	
You each receive a stack of 20 theme cards. Choose your personal top five themes to answer the following question: Which developments over the past 10 years have had the greatest impact on HIC implementation on your ward? Then chose one theme card that you think was least of influence on HIC implementation at your ward. Motivate your top five themes and your least of influence theme.	
Personnel	Healing environment
From control to contact	Training
Safety	Budget
Cooperation	Expertise by experience
Coercion	Working methodically
Contact with relatives	An empty ICU is a good ICU^a^
Cooperation with outpatient care	Demographical aspects
Recovery at home	Network psychiatry
Addiction	COVID‐19^b^
Aggression	—[own addition]

^a^ Intensive Care Unit: a physical space within the high and intensive care ward where staff can move to with a patient to provide more intensive, one‐on‐one care.

^b^ Coronavirus Disease 2019: an infectious disease caused by the SARS‐CoV‐2 virus. The pandemic, declared by the World Health Organization in March 2020, has had significant global impacts on public health, including widespread psychological and social consequences.

**TABLE 3:** Topic guide national focus groups.

National focus group number	Topics and probes
1	Care at the ward Personnel Cooperation with family
2	Working methodically Cooperation with outpatient care
3	Safety Coercion Aggression Evaluation of aggression and coercion
1, 2 and 3	What have we learned?
	How to move forward?
	What does this say about your ward culture?

### 3.4 Analysis

Preliminary analysis of the group interviews involved thematic analysis to identify recurring themes and comparative case analysis to identify patterns and cross‐group differences (Gray 2018). Then, thorough thematic analysis (Gray 2018) was carried out on the institutional interviews using MAXQDA 2022 (VERBI Software 2021). We used a combination of deductive coding, guided by an established code tree (Table 2), and open coding, which allowed for the addition of new codes that emerged from the data (Fereday & Muir‐Cochrane 2006). To enhance confirmability, we employed investigator triangulation (Holloway and Galvin 2017). IdJ first coded three interviews, whereupon LvM, SG, YV , and one other consulted researcher each coded two interviews separately, using the existing coding tree. Afterwards, they discussed any overlap and new codes with IdJ before collectively determining which codes should be either adapted or added to the coding tree. Finally, codes were clustered into themes and refined through discussion until consensus was reached. We focussed on participants' expressed content and meaning rather than analysing numerical rankings. Subsequently, national focus groups were coded similarly by IdJ, using the themes and codes found in the group interviews. The data from the focus groups confirmed the codes and themes identified in the earlier interview data, further refining and solidifying the core themes without leading to the emergence of new themes, indicating that data saturation had been reached (Gray 2018). We identified five main themes, from which four emerged from the deductive categories and one was altered from ‘personnel’ to ‘staff turnover’.

## 4 Results

This section presents the findings of the study. Based on the analysis of the data, we identified five themes: staff turnover, coercion, collaboration with outpatient care, working methodically and the move from control to contact.

### 4.1 Staff Turnover

The most prominent development within the prior 10‐year period concerned staff turnover that had led to both positive and negative effects for HIC wards, where the negative predominated. On one hand, staff who struggled to adapt to the shift towards the HIC model left the wards, making room for (new) staff who embraced the HIC model without bias. On the other hand, staff turnover led to an imbalance between experienced, familiar staff and less experienced, unfamiliar staff. The latter were mainly temporary workers and younger employees who had just completed their training. This imbalance led to both an unsafe feeling amongst staff and more staff turnover once again.

I believe the main issue at the moment is the quality of staff, as many new hires are recent graduates or temporary workers. With such high turnover, there isn't enough time to properly train everyone. For inexperienced individuals, this environment can feel very unsafe, leading many to choose a different job. Nurse

The significant rise in temporary workers posed a problem as they were generally unfamiliar with the HIC method, patients and the team. This, in turn, increased the workload of permanent staff and led to escalations with patients and discontinuity of care. The participants stressed the importance of trust and familiarity within a team, especially within an HIC ward where quick action is crucial for everyone's safety.

“Of course, when I'm alone with three temporary workers and a patient goes crazy, I won't make contact the entire day, I will prioritise safety [through control]. However, when I work with these colleagues [pointing at permanent colleagues], we work together to find a way to calm the patient down [without control] because we believe we can manage”. Nurse

Participants noted that the national shortage of psychiatrists caused discontinuity in ward/HIC policy and care, leading to missed interventions, longer stays and increased coercion. Despite managers' efforts to meet Full‐Time Equivalent (FTE) requirements, the lack of a permanent team hindered HIC effectiveness. They recommended improving staff retention, support, salaries and working conditions and discontinuing temporary hires. Participants saw positive effects from creating a welcoming environment for new employees, including those in support roles like care security guards, activity counsellors and peer counsellors, who improved safety and reduced workload. However, their roles must be well integrated into the team.

In the beginning, I felt unsafe a few times because there was a lot of uncertainty about my role. Colleagues acknowledged this by saying things like, “Okay, we're really trying to figure out: what are you doing here?” The feeling of not being supported by my colleagues made me think: “if I press the alarm now, things will probably go wrong because they have no idea what is happening.” There was a sense of being ignored, as if I wasn't there. Peer counsellor

### 4.2 Coercion

Another development that all the participants felt HIC had changed for the better was the reduction of coercion over time. Implementing HIC had increased their awareness of both their own use of coercion and how more could be achieved by using less coercion, such as applying the ‘first five minutes’ intervention. This intervention entails various actions to establish a good first encounter with the patient, both verbal and non‐verbal.

We have become more aware of our approach. Previously, anyone who came in off the street would be placed in the seclusion room first. Now, with the ‘first five minutes’ method, we start by assessing the person, and if necessary, begin in the ICU. From there, we evaluate the situation and proceed accordingly. Nurse

In addition, the Dutch Compulsory Care Act facilitated the initiation of treatment within HIC from the outset. This, in turn, improved the quality and efficiency of care provision as well as the atmosphere within wards. However, many of the participants opined that coercion, particularly seclusion, was still used excessively, despite its shorter duration in recent times and that stepping up care was often overlooked due to staff shortages and a lack of facilities. One care manager suggested that the issue was altogether more complex.

I see seclusion as a symptom of other processes like: how is the collaboration between outpatient and inpatient care, and between treatment team and nursing team? I think if you have more trust, more time spent together, then many seclusions that are happening now, out of panic, fear, unsafety, or sometimes incompetence, could be prevented. Care Manager

Three wards had eliminated the use of seclusion. This had prompted heated debate during the national focus groups, as the majority of participants—reflecting prevailing staff attitudes—maintained that seclusion was essential for ensuring safety, particularly with highly aggressive, often substance‐affected patients, and for reducing stimuli. Moreover, they believed that, when applied efficiently and proportionately, seclusion was a preferable alternative, beyond for example, forced medication.

I truly believe it is an illusion to think that seclusion can be fully eliminated. I'm not sure what is more humane—holding someone down on the floor in their room with eight people and forcibly administering medication or placing them in an seclusion room for a day without anyone physically intervening. Nurse

All of the wards acknowledged that they did not systematically evaluate coercive measures. Typically, only major incidents were evaluated by the team, who were generally more focused upon intervention than prevention, whilst patients were mostly not involved in the evaluation.

### 4.3 Collaboration With Outpatient Care

Participants who were positive about their collaboration with outpatient teams had taken them along with HIC implementation from the outset and had made clear arrangements concerning expectations, admission, and discharge criteria, as well as what an HIC can and cannot do. Moreover, some wards had appointed admission coordinators to safeguard these arrangements.

As a municipality, you also have the responsibility for your citizens, and the health insurer also has a duty of care in that regard, so these conversations have taken place much more in recent years, in defining that boundary, saying: “Yes, this patient is going to be discharged tomorrow or the day after, so I need you for this, and then you can see what you, as the other party, are going to do.” Admission Coordinator

Participants with negative experiences of outpatient care were unaware of any coordination with external teams and felt frustrated by their lack of involvement, refusal to (re)admit patients, or limited capacity. This conflicted with the HIC principle of ‘recovery at home,’ which sees inpatient care as crisis stabilisation, with real recovery starting at home. Similar issues arose with sheltered housing and forensic facilities, which were often unavailable, frustrating both staff and patients

The complex patients who cannot be placed anywhere else stay longer because outpatient care says: “They really can't go home.” Sheltered housing says: “No, we're definitely not taking them back.” So, you have to keep them, and these are the big disruptors at a ward that you're just keeping afloat until a suitable location becomes available. Coordinating nurse

On the whole, participants were very positive about the emergence of Intensive Home Treatment Teams, who were able to both set up acute intensive care at home quickly, even outside of office hours. Care Coordination Meetings (CCMs), which are regular meetings to collaboratively establish admission goals with the patient, relatives, outpatient care providers, and HIC staff, fostered collaboration, improved admission efficiency and reduced admission time.

That [Achieving success through the CCM] was with a patient who arrived with severe suicidal behaviour over the weekend. On Monday, a CCM took place right away, involving both outpatient care and a supportive care farm. In that CCM, everything came together, and it resulted in a 6‐day admission. So, it is possible, and it's great to see that happen. Nurse

### 4.4 Working Methodically

While most teams actively worked with HIC methods, some felt they did not have to work methodically in order to work according to the HIC vision. However, several participants noted that some colleagues—mainly physicians—had never read the HIC workbook and lacked both knowledge and commitment. They emphasised the need for reflection, awareness and a clear vision to strengthen model fidelity.

Transferring the [HIC] vision to new employees, that's what we started to invest in. Our team has messed around for years and is now becoming stable whilst we have more [staff] turnover than ever. But because we are finally getting on the same page by having a vision, a plan and an aim, that helps a lot [in safeguarding HIC]. Nurse

Whilst some teams had prioritised training staff over time, several others reported an ongoing lack of adequate training, particularly in drug addiction and intellectual disabilities. The HIC course, launched in 2018, improved participants' understanding of the HIC method and facilitated exchange of knowledge and experiences, which many participants aspired. Moreover, they expressed the need for a practical workbook to instruct professionals in how to apply HIC methods. All this should lead to greater uniformity of practice across HIC wards which was deemed to be essential for reliably implementing policies. For example, policies to promote patient autonomy which require alignment with outpatient care teams and the broader system, that begins with a uniformed HIC policy.

I believe that we, as HICs, need to speak a unified language nationwide. We each still have our distinct ways of working, which really shouldn't be the case in such a specialised field. Coordinating nurse

In a considerable number of wards we visited, it appeared that the healing environment and stepped care elements were not being implemented as intended. Comfort rooms were locked, dirty or repurposed, and ICUs were either locked, occupied or constantly staffed, whilst the HIC model suggests unmanned ICUs for nurse accompaniment. Furthermore, ward conditions varied greatly, however, new construction alone was not viewed to be a panacea.

I think it's quite impressive that we've managed to implement the HIC methodology in a ward that doesn't ideally align with the HIC model, and that perhaps HIC work itself is more important than the environment. You can have a great physical environment, but if you continue to control and aren't collaborating, I think you're in a much worse position than if you're in an old building with an appropriate way of working. Nurse

### 4.5 From Control to Contact

A positive development cited by all of the participants concerned the move from control to contact‐based care, which changed practice as a whole. The participants routinely described HIC as a ‘cooperation model’ with less emphasis being placed upon repression and more upon treatment (see also ‘coercion’). The participants thought that rules and controlling ways of working often led to conflict and aggression, whereas being creative and thinking outside the box reduced aggression and increased job satisfaction.

These [strict ward] rules simply don't work. At the forensic HIC, my colleagues were excessively focused on every minor rule and were very controlling. This led to conflicts daily. However, when you engage in normal, respectful contact, there's no issue at all. Former forensic nurse

Only a few participants thought that control was first required to create a therapeutic and safe environment, prior to making contact. The participants had come to learn that they needed trust to both accept and dare to take risks, but also, as one physician described, to ‘carry risks’, as opposed to avoiding them. However, as shown in ‘staff turnover’, working with familiar colleagues was regarded as being a prerequisite for making contact. Another thing that the participants had come to learn over time was to be more present and available to patients.

When I first started working here, we spent most of our time in the office and rarely on the ward. Over time, the office was phased out, and we began spending more and more time with the group. Being visible and present made it easier and faster to make contact. We became more aware of ward dynamics, which in turn led to a reduction in aggression. Nurse

Despite removing offices and introducing laptops, it was observed that most teams still clustered together behind open desks rather than being with the group. Physicians were mostly found in their offices outside of the wards. Participants concluded that changing practices is possible but needs time and behavioural change.

You can simply choose not to use the office anymore, but it took time. It also required behavioural changes from colleagues and letting go of a sense of security, as the office had been a personal space where one could retreat. Initially, this led to some resistance. However, the feedback I've received has been overwhelmingly positive. Nurse

## 5 Discussion

This study shows that, from professionals' perspectives, the most influential developments over the prior 10‐year period within their HIC (accurate) work occurred within five domains: staff turnover, coercion, collaboration with outpatient care, working methodically and the move from control to contact. Our analysis demonstrated both strong alignment and points of divergence between wards with respect to these developments. The themes identified in this study align with the implementation science framework, addressing the persistent evidence‐practice gap in clinical settings (Handley et al. 2016). According to Handley et al. (2016), there are three key strategies to close this gap: (1) behaviour change, (2) engagement of stakeholders and (3) flexibility and often non‐linear approaches. We will further discuss our findings and touch upon their relation to these key strategies.

In accordance with van Melle et al. (2021a), staff shortages were found to be the main issue in HIC implementation. Statistical evidence shows that between 2018 and 2022, staff shortages doubled and are projected to nearly triple within the next decade (Boumans et al. 2023). However, professionals expressed urgent concern over staff quality, emphasising that expertise and competency outweigh sheer numbers. Teams with fewer, but experienced familiar staff reported higher safety and less coercion compared to those with more, temporary or inexperienced staff. This relates to the first key element of implementation science, namely that evidence should be translated into practice (Handley et al. 2016). Though evidence suggests that experienced, consistent staffing leads to better outcomes, the practice gap exists because individuals or organisations prioritize quantity (more staff) over quality (competent, familiair staff). To illustrate, the addition of temporary staff in our study actually increased the workload, while 48% of mental health organisations in the Netherlands increased staff numbers as a strategy to reduce workload (de Rooij and Raateland 2023). We suggest switching focus from solely increasing numbers towards hiring the right people with permanent contracts. According to Woltmann et al. (2008), staff turnover can be used to improve hiring practices and realign policies to create better teams. However, more staff consistency and better doctor‐patient ratios improve outcomes and reduce costs (Brandt et al. 2016). We recommend further research into staff retention and acquisition, along with recommending that HIC wards explore alternative ways to ensure continuity of care, such as hiring nurse specialists who can provide high‐quality care in a timely and cost‐effective way (Fisher 2005).

Although there was a perceived reduction in coercion over time, people continued to believe that compulsory care was common and necessary when safety was compromised. According to the first key principle of implementation science, staff attitudes are crucial, as they directly impact the adoption of evidence‐based practices. Even proven interventions may be poorly implemented if met with scepticism, fear of change or perceived irrelevance (Handley et al. 2016). In our study, aggression and staff shortages were key barriers to further reducing compulsory care, unlike Yersin et al.'s findings (2024). In a recent survey of 116 mental health professionals, Yersin et al. (2024) revealed most believed seclusion had a therapeutic role, which partly aligned with our research as some people believed that seclusion helped to reduce stimuli. We therefore recommend research into the specific barriers to reducing seclusion in HIC settings and the use of recommended alternatives, as well as case studies focused specifically on seclusion. Practically, we agree with Tingleff et al. (2017) that more training on empathetic behaviour, communication skills and sensitivity to patients' perspectives at all stages (before, during and after coercion) may be more effective than the usual de‐escalation trainings. Structural evaluations of coercion, including patient debriefings, should be standard in order to both minimise emotional impact and prevent future crises (Georgieva et al. 2012; van Melle et al. 2021b).

At the same time, our results indicate that safety has improved over time, with contact‐based work enhancing safety more than control‐based approaches. This ‘positive risk‐taking’ requires professionals and patients to collaboratively assess risks and benefits (Morgan 2004). Vandewalle et al. (2018) found that both young nurses and/or those working in closed wards often felt reluctant to engage in positive risk‐taking, and that they required additional education and managerial support. We posit that similar support is required for implementing best practices such as removing offices and promoting presence. Understanding the barriers of change are needed to facilitate this behavioural change where the COM‐B (Capability, Opportunity and Motivation) model offers a useful approach (Handley et al. 2016).

Despite the HIC model's emphasis on collaboration with outpatient care, we found that collaboration was often poorly established, with little alignment or agreement amongst parties. This reflects the second key element of implementation science, which stresses the importance of engaging diverse stakeholders for effective translation and sustained improvements. Similar to our findings, many initiatives to improve healthcare quality have been implemented without direct input from the affected individuals or communities (Handley et al. 2016). Conducting research on the perspectives of outpatient care teams and their collaboration is crucial to better align HIC practices with outpatient care and increase job satisfaction (Wright et al. 2016). A key policy priority should be to address the ethical dilemma of the lack of resources, namely, who is responsible when patients are ready to be discharged or when patients do not meet admission criteria but are in need of help? Our study, supported by Stewart et al. (2012), suggests that admission coordinators may play a key role in resolving these issues.

Moreover, our study revealed considerable variation in applying the HIC model, underscoring the ongoing effort required from teams to create more uniformity of practice and closing the gap between evidence and practice (Handley et al. 2016). Coordinated strategies and clearer guidance on best practices, for example in the form of a more practical workbook, may close this gap, promote uniformity and improve care quality. Communities of practice may stimulate this process by fostering peer learning (Gerritsen et al. 2021).

### 5.1 Strengths and Limitations

A key strength of this study is the diverse sample of wards and participants. This allowed us to underscore differences and similarities across the wards. The initial institutional focus groups created a safe environment for participants to share their experiences (Holloway and Galvin 2017). The national focus groups fostered idea‐sharing and enhanced reliability by enabling cross‐checking of the previous interview results and establishing data saturation (Gray 2018). Moreover, this study aligns with the third key element of implementation science—the cyclical, long‐term approach suited to real‐world settings (Handley et al. 2016). One potential limitation of the study is recall bias, in as far as the participants were asked to reflect upon 10 years of practice, which may have skewed their perceptions of the current or past situation (Holloway and Galvin 2017). However, the amount of group interviews conducted was adequate for data validation. Finally, our study focused solely on inpatient professionals, excluding community‐based stakeholders who are closely linked to HIC developments and could offer valuable insights. Future research should include their perspectives.

## 6 Relevance to Clinical Practice

Although this article focuses on HIC, the findings and insights presented are broadly applicable and can be generalised to many clinical practices internationally that focus on implementing strategies to improve quality of care, offering valuable perspectives for mental health care policy makers and providers worldwide. The findings emphasise the need for continued attention to staff retention and recruitment, alongside the reduction of coercive measures, such as seclusion, through structured evaluation. Moreover, it underscores the importance of fostering better collaboration between inpatient and outpatient care, as well as ensuring ongoing training, reflection and strong management support. By providing tools to navigate these challenges, this study encourages HIC wards to refine their practices and emphasises the value of mutual learning through collaboration between wards. These insights offer critical guidance for improving patient care, ensuring a more sustainable and effective implementation of the HIC model within mental health settings.

## 7 Conclusion

Over the past decade, several developments have enhanced HIC practice as well as impeded its further development. This study underscores that having a permanent team, strong alignment with outpatient care, and awareness about coercion, reflection and behavioural change serves to improve HIC practice. Enhanced sharing of practices should help to promote greater uniformity of practice amongst HIC wards. Our study puts forward several recommendations for further research, which could contribute towards advancing knowledge and improving practices within the field of acute mental health care.

## Author Contributions

Data collection was conducted by IdJ, LvM, SG and YV. IdJ performed the initial analysis and drafted the manuscript. LvM, SG and YV also contributed to the data analysis, and all authors contributed to and revised the manuscript. All authors meet the authorship criteria according to the latest guidelines of the International Committee of Medical Journal Editors and contributed to and approved the final version of the manuscript.

## Ethics Statement

This study was conducted and reported in line with the COREQ guidelines for reporting qualitative research (Tong et al. 2007). Two authors were employed at two participating HIC institutions; however, they were not part of the HIC team involved in the study. Their roles at the institution did not influence the data collection process or the interactions with the participants. The Medical Ethics Review Committee of the Amsterdam University Medical Center declared that this study is a non‐Law Medical‐Research study with people [niet‐WMO] with reference number 2022.0625 (VU, 2022). The Amsterdam Public Health Research Institute embedded this study with reference number SQC20222‐047.

## Consent

All participants were informed of the study's purpose and their voluntary participation; informed consent was obtained for participation and publication of this study. Moreover, confidentiality was ensured during the group interviews and focus groups, and we strived for equal opportunity for all participants to share their experiences.

## Conflicts of Interest

The authors declare no conflicts of interest.

## Data Availability

The data that support the findings of this study are available on request from the corresponding author. The data are not publicly available due to privacy or ethical restrictions.
